# Viral Hepatitis and Liver Diseases in Mongolia

**DOI:** 10.5005/jp-journals-10018-1215

**Published:** 2017-05-05

**Authors:** Oidov Baatarkhuu, G Uugantsetseg, D Munkh-Orshikh, N Naranzul, S Badamjav, D Tserendagva, J Amarsanaa, Kim Do Young

**Affiliations:** 1 Department of Infectious Diseases, Mongolian National University of Medical Sciences; 2 Mongolian Association for the Study of Liver Diseases; 3 Department of Internal Medicine, Mongolian National University of Medical Sciences; 4 International School of Mongolian Traditional Medicine, Mongolian National University of Medical Sciences; 5 Department of Internal Medicine, Yonsei University College of Medicine, Korea

**Keywords:** Hepatitis A virus, Hepatitis B virus, Hepatitis C virus, Hepatitis D virus, Hepatocellular carcinoma.

## Abstract

Mongolia is known for its high endemicity for viral hepatitis. Previous studies report that the seroprevalence of hepatitis B virus (HBV) is 11.8% (178/1,512) among the unvaccinated population in 13 provinces and Ulaanbaatar city. The serosurvey of adults (>20 years of age) conducted during 2013 among persons in four provinces and in Ulaanbaatar showed that the overall prevalence of hepatitis B surface antigen (HBsAg) positivity was 10.6%. The overall prevalence of anti-hepatitis C virus (HCV) and HCV ribonucleic acid among 1,512 apparently healthy subjects was 15.6% (236/1,512) and 11.0% (167/1,512) respectively.

In a previous study, we reported on the prevalence of HBV, HDV, and HCV infections in 110 consecutive patients presenting with acute hepatitis at eight city hospitals in Ulaanbaatar. In that study, 16.4, 32.7, 6.4, 1.8, and 27.3% of the patients were diagnosed as having acute hepatitis due to hepatitis A, B, C, HBV/HDV coinfection, and superinfection respectively. In the current study (2012-2014), results show that acute hepatitis A, B, C, and D was diagnosed in 47.9, 40.7, 5.3, and 9% respectively.

Chronic HBV and HCV infections, which are associated with cancer and cirrhosis respectively, are responsible for 95% of liver cancers in Mongolia. The most common etiology for hepatocellular carcinoma was HCV infection (n = 89, 45.6%), followed by HBV infection (n = 67, 34.4%).

**How to cite this article:** Baatarkhuu O, Uugantsetseg G, Munkh-Orshikh D, Naranzul N, Badamjav S, Tserendagva D, Amarsanaa J, Young KD. Viral Hepatitis and Liver Diseases in Mongolia. Euroasian J Hepato-Gastroenterol 2017;7(1):68-72.

## INTRODUCTION

Mongolia is a developing country located in Central Asia. The population of Mongolia as of July 2007 is estimated at around just 2.9 million people, ranking 138th in the world in terms of population. With a relatively young population structure, 32.7% are under the age of 15 years and only 3.5% are over 65 years of age. Approximately 40% of the population continues to live a traditional nomadic or seminomadic lifestyle.^1^

Mongolia is an area in which viral hepatitis B and C are highly prevalent. Hepatocellular carcinoma (HCC) is the most common cancer in Mongolia, occurring at a rate of 54.1 cases in 100,000 people. The relative importance of hepatitis B virus (HBV) and hepatitis C virus (HCV) infections in HCC etiology is known to vary greatly from one part of the world to another. Although surveillance tests, such as ultrasonography, are important in early detection of HCC and in improving the survival, it is hard to perform surveillance tests due to the scarcity of equipment in developing countries, such as Mongolia. Therefore, observation on the clinical characteristics and course of Mongolian HCC patients would be of help to understand the importance of HCC surveillance, prevention, and appropriate treatment.

## VIRAL HEPATITIS A AND E

The prevalence of hepatitis virus infections among 110 consecutive patients (16-48 years) with acute hepatitis in Mongolia, HAV (IgM-positive) was diagnosed in 18 patients (16.4%), had detectable HAV ribonucleic acid (RNA).^1^ In the current study (2012-2014), results show that acute hepatitis A was diagnosed in 47.9%.^2,3^

**Graph 1:**
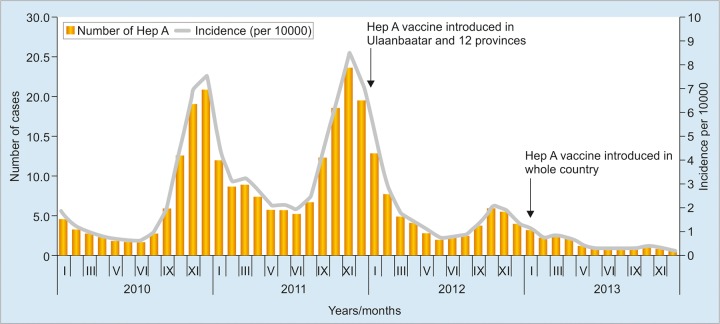
Acute jaundice from HAV and introduction of HAV immunization in infancy *(Source:* NCCD 2014)

However, HAV seroprevalence among elementary schoolchildren 7 to 12 years of age in this study was 84.2% (438 out of 520).^4^

Hepatitis A virus was the most common cause of acute jaundice reported until immunization for HAV was introduced among infants in 2012 (Graph 1). The lack of chronicity and seroprotection following HAV infection, combined with the introduction of the HAV vaccine, resulted in an increasing proportion of the population being immune to HAV infection.

In 2012, domestically funded HAV immunization was introduced in infant vaccination schedules (at 1.2 and 2 years), which reduced the proportion of acute jaundice cases related to HAV infection, and HAV has seasonal peaks in Mongolia. In 2010, the peak was ~8/10,000 in December. The 2011 peak was 9/10,000 in November. In 2013, there was no seasonal peak, with the HAV rate highest in January at 1/10,000 and reducing as the year progressed.^5^

Vaccine coverage was 92.3% in 2012 and 95.9% in 2013. It is plausible that infant vaccination has resulted in a reduced pool of infectious individuals and impacted acute HAV rates at a population level.

The seroprevalence of hepatitis E virus (HEV) immunoglobulin (Ig)G is high among Mongolian adults above 30 years of age, and is estimated at 12%. Seroprevalence in children is very low (0.8%).^4^ Human disease from HEV has not been recorded in Mongolia.

## HBV, HCV, AND HDV INFECTION

In a previous study, we reported on the prevalence of HBV, HDV, and HCV infections in 110 consecutive patients presenting with acute hepatitis at eight city hospitals in Ulaanbaatar. In that study, 32.7, 6.4, 1.8, and 27.3% of the patients were diagnosed as having acute hepatitis due to hepatitis B, C, HBV/HDV coinfection, and superinfection respectively.^2^

The current study population consisted of 624 patients clinically diagnosed with acute hepatitis during the period of January 2011 to December 2013 in Ulaanbaatar, Mongolia. A time trend analysis investigating the change in acute HAV, HBV, HCV, and HDV incidence among the cohort with respect to a previous published study was undertaken. Acute hepatitis B, C, and D were diagnosed in 40.7, 5.3, and 14.7% respectively. Notably, 21% of the cohort had a dual infection. Nonsterile medical-related procedures were the main identified risk factor. Unfortunately, there was only small but nonsignificant decline in the incidence of acute HBV and HCV infections over the last decade, though there was a significant decline in HDV incidence.^3^

There have been a number of seroprevalence surveys of HBV infection among children in Mongolia, conducted to assess the effectiveness of the HBV immunization program. A national community-based, multistage cluster sample survey in 2009 to 2010 (n = 5,894) estimated the seroprevalence of hepatitis B surface antigen (HBsAg) in 4- to 6-year-old children at 0.53%, verifying that Mongolia had achieved the 2012 regional milestone of an HBsAg prevalence of less than 2% among 5-year-old children.^4^

Previous studies report that the seroprevalence of HBV is 11.8% (178/1,512) among the apparently healthy population in 13 provinces and Ulaanbaatar city. The prevalence was similarly high across age groups ranging from the 20s through the 60s. Prevalence was less among those 60 years and above. In Ulaanbaatar, the prevalence was estimated to be 9.3%.^6^

**Graph 2:**
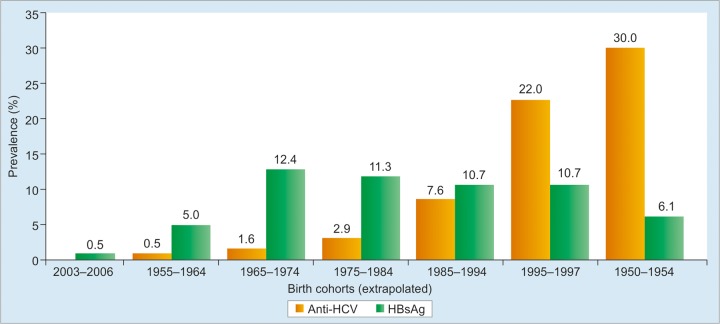
Prevalence of anti-HCV antibody and HBsAg by age cohort in Mongolia *(Sources:* Baatarkhuu et al; Dashtseren et al; Davaalkham et al)^1,4,7,8^

Among men who have sex with men (MSM), the prevalence of HBsAg has been estimated at 14%, and the prevalence of HBV coinfection among human immunodeficiency virus (HlV)-infected MSM is 8%. However, there are fewer than 200 HIV cases identified in the country.^5^

The overall prevalence of anti-HCV and HCV RNA among the study subjects was 15.6% (236/1,512) and 11.0% (167/1,512) respectively (Graph 2). The prevalence of HCV infection was above 16.7% in rural provinces, such as Bayankhongor, Arkhangai, and Dornod. The graphs were significantly higher than those in rural provinces, such as Uvurkhangai or Umnugovi (p < 0.01). The positive rate of anti-HCV and HCV RNA in Ulaanbaatar was 13.7% (41/300) and 9.7% (29/300) respectively. Among 167 HCV RNA-positive samples, 165 (98.8%) were classified into genotype 1b and the remaining two were genotype 2a. The HCV genotype 2a isolates were obtained from a 48-year-old female and a 56-year-old male who resided in urban areas.^1^ In general, prevalence is estimated to be higher in rural areas, particularly in the Northern provinces, and lower in Ulaanbaatar and the Southern provinces. In a study conducted in 2002 to 2005,^5^ the estimated prevalence ranged from 7.3% in Uvurkhangai to 18% in Khuvsgul in the north, bordering Russia. The prevalence in Ulaanbaatar was close to the country mean in both the surveys. The highest anti-HCV prevalence was seen in three rural aimags in 2004 to 2005, namely Dormod (18.2%) in the eastern region, and in two Khangai region aimags (Bayankhongor 17.2%, Arkhangai 16.7%). Of the four aimags included in the 2013 survey, Zavkhan (western region) and Uvurkhangai (Khangai region) had a relatively high prevalence.^1^

Among adults, the prevalence of anti-HCV antibody increases with age, with the highest prevalence in both population-based studies seen among persons in birth cohorts from the mid-1950s and earlier (i.e., persons who are currently 60 years and older), and lowest among persons in the birth cohorts from about 1990 onward (i.e., persons who are currently in their mid-20s and younger). A nationwide population-based sample of elementary schoolchildren (ages 7-10 years) conducted in 2004 (10) suggests that persons in the birth cohort of approximately 1994 to 1997 (currently aged 17-20 years) have a very low anti-HCV prevalence (<1%).^5^

Hepatitis D infection is highly endemic among individuals with chronic HBV infection in Mongolia. The likely reason is the association between high HBV prevalence, the existence of HDV in the community, and blood-borne virus transmission in health and nonhealth settings over the past decades. The prevalence of antibody to HDV (anti-HDV) among HBsAg-positive individuals, indicating likely HBV/HDV coinfection, ranged from 41 to 67% in the two major seroepidemiological studies. The National Enterovirus and Hepatitis Laboratory reported that in 2013, 45% of HBsAg-positive samples were also anti-HDV positive, while 30% were HDV IgM positive. The HBV/HDV coinfection is associated with more rapid progression of liver disease than is seen in HBV monoinfection. For example, a 2005 study of patients with chronic liver disease reported a prevalence of HDV RNA of 39% among patients with chronic hepatitis, 51% among those with cirrhosis, and 80% among those with HCC.^6,7^ These data are consistent with observations of HBV/HDV coinfection in Mongolia.

## HEPATOCELLULAR CARCINOMA

Hepatocellular carcinoma is a prevalent and usually fatal cancer that mostly affects persons in developing countries. Since HBV and HCV infection are the predominant causes of HCC, the incidence of HCC is parallel with the incidence of this chronic viral hepatitis. In particular, HBV appears to be the most important etiological agent for HCC in South-East Asia including Korea and China, whereas HCV is the most common cause of HCC in the USA and Japan. Hepatocellular carcinoma is the most common cancer in Mongolia, occurring at a rate of 54.1 cases in 100,000 people.^8,9^

In 2013, there were 1,967 newly diagnosed liver cancer cases and 1,578 deaths due to liver cancer. Approximately 11% of cases were diagnosed with histological confirmation, 14% by clinical examination, the remainder by imaging or other methods. Over 80% of all cases were diagnosed in the late stages: 4% in stage I, 14.6% stage II, 48% stage III, and 33.2% in stage IV.^5^

The most common etiology for HCC was HCV infection (n = 89, 45.6%), followed by HBV infection (n = 67, 34.4%). The mean tumor diameter was 6.0 ± 2.6 cm. Only 29 (14.9%) patients had a single lesion, while 39 (20.2%) had lesions. Extrahepatic metastasis to lung (n = 23), bone (n = 10), and lymph node (n = 3) were detected in 36 (18.5%) patients. Most patients had advanced HCC - 88 (45.1%) in stage III and 57 (29.2%) in stage IV. Surgical resection was performed in 27 (13.8%) patients, radiofrequency ablation in 23 (11.8%) and transarterial chemoembolization in 107 (54.9%). When all the patients were categorized as "treated" (n = 156) and "not treated" (n = 39), the 3-year survival was significantly lower in the "not treated" group than in the "treated" group (11 *vs* 0%, p=0.001).^8,9^

Tumor diameter (3< *vs* ≥3 cm), extrahepatic metastasis, tumor/node/metastasis stage (I/II *vs* III/IV), and treatment (or supportive care) were selected as independent predictors for survival.

A significant proportion of patients presented with symptoms, such as fatigue (78%), weight loss (76%), abdominal pain (15%), and lump (18%). Among all HCC patients, 38 (19.5%) and 51 (26.2%) patients had a history of blood transfusion and tattooing respectively. Out of 195 HCC patients, only 32 (16%) had pathologically proven HCCs and were diagnosed by imaging and tumor marker. There was history suggestive of acute hepatitis in 120 (61.5%) and of chronic hepatitis in 102 (52.3%) patients.^8,9^

## CONCLUSION

In Mongolia, there is an urgent need to control the highly prevalent viral hepatitis B and C to decrease the incidence of HCC. Improvement of surgical, interventional, and medical techniques is also required to manage patients with HCC in Mongolia. Further efforts to search for risk factors for hepatitis virus infection in the general population and among patients with acute or chronic liver disease would be useful for elucidating unapparent modes of transmission and for preventing the spread of these hepatitis viruses among people living in Mongolia. With the implementation in 1995 of a national policy mandating single-use syringes in health care facilities, the risk of injection-associated transmission of HCV, HBV, and HDV in Mongolia is likely to have decreased significantly. However, assurance of effective procedures for disinfection and sterilization of medical equipment is more challenging, and there are persistent problems associated with reuse of medical instruments and other equipment intended for single use for a number of reasons. These include budgetary constraints, stock-outs, and competing demands on the health care system; challenges in autoclave maintenance; as well as inadequate numbers of nurses, and insufficient personnel and training to conduct regulatory inspections. There is particular concern about the potential role of dental clinics, mostly private, in maintaining the transmission of hepatitis viruses, though there are scant data to support or refute this claim. These concerns are not unique to Mongolia and other low- and middle-income countries, but especially high prevalence of viremia for HBV, HCV, and HDV among the population amplifies the risks associated with breaches in safe injection practices and infection control. There is some limited evidence suggesting that health care workers have a higher prevalence of blood-borne infections, related in part to a historically high rate of needle-stick injuries; in 2013, the Ministry of Health and Sports initiated a program for systematic HBV immunization of health care workers and students in the health professions, which had made great progress by late 2014. Medical waste management and disposal is also an issue; currently, most sharps waste is incinerated and buried in landfills. There is concern about historical beliefs and practices among both consumers and providers of healthcare, which resulted in very high rates of unnecessary medical injections as well as medically unsupervised injections of over-the-counter antibiotics and vitamins received in the home. The health care system has also historically promoted hospitalization (over outpatient care) for large numbers of patients, straining resources and increasing the potential for nosocomial exposures.

## References

[1] Baatarkhuu O, Kim DY, Ahn SH, Nymadawa P, Dahgwahdorj Y, Shagdarsuren M, Park JY, Choi JW, Oyunbileg J, Oyunsuren T (2008). Prevalence and genotype distribution of hepatitis C virus among apparently healthy individuals in Mongolia: a population-based nationwide study. Liver Int.

[2] Tsatsralt-Od B, Takahashi M, Endo K, Buyankhuu O, Baatarkhuu O, Nishizawa T, Okamoto H (2006). Infection with hepatitis A, B, C, and delta viruses among patients with acute hepatitis in Mongolia. J Med Virol.

[3] Munkh-Orshih D., Eslam M., Kim DY., Oyungerel R., Baigali B., Dagvadorj Y., George J., Ahn SH., Han KH., Baatarkhuu O. (2016). Changing incidence of acute hepatitis A, B, C and D in Mongolia. Abstract book..

[4] Davaalkham D, Enkhoyun T, Takahashi M, Nakamura Y, Okamoto H, Hepatitis A (2009). E virus infections among children in Mongolia. Am J Trop Med Hyg.

[5] Walsh N, Nick W Geoffrey Beckett, Ann Chao, Ying-Ru Lo Viral hepatitis in Mongolia: situation and response. http://www.wpro.who.int/hepatitis/hepatitis_resource_publica-tion/viral_hepatitis_mongolia_2015/en/publication.

[6] (2012). Oidov Baatarkhuu, Bira Tsatsralt-Od, Chuluunbaatar Bolormaa, Geleg Tsetsegmaa, Bat-Ulzii Saruul, Tooloi Alimaa, Jadambaa Bayarsaikhan, Osorjin Buynkhuu, Manaljav Shagdarsuren, Yagaanbuyant Dagvadorj. Prevalence and genotype distribution of dual or triple infection of hepatitis B,C and D viruses among patients with chronic liver diseases of Mongolia. Hepatol Int.

[7] Dashtseren B, Bold B, Dashdorj N, Yagaanbuyant D (2014). P 29: epidemiological study of prevalence and risk factors for HBV among apparently healthy Mongolians. J Viral Hepat.

[8] Baatarkhuu O, Kim DY, Nymadawa P, Kim SU, Han KH, Amarsanaa J, Gonchigsuren D, Sanduijav R, Lkhagvasuren Z, Khorolsuren N (2012). Clinical features and prognosis of hepatocellular carcinoma in Mongolia: a multicentre study. Hepatol Int.

[9] Baatarkhuu O, Kim DY, Bat-Ireedui P, Han KH (2011). Current situation of hepatocellular carcinoma in Mongolia. Oncology.

